# Candidate insect vectors of apple proliferation in Northwest Spain

**DOI:** 10.1186/s40064-016-2907-9

**Published:** 2016-08-02

**Authors:** Marcos Miñarro, Aitor Somoano, Aránzazu Moreno, Rocío Rosa García

**Affiliations:** 1 Servicio Regional de Investigación y Desarrollo Agroalimentario (SERIDA), Apdo. 13, E-33300 Villaviciosa, Asturias Spain; 2 Instituto de Ciencias Agrarias-CSIC, Madrid, Spain

**Keywords:** *Cacopsylla melanoneura*, *Cacopsylla picta*, *Candidatus* Phytoplasma mali, *Ctenarytaina*, Disease transmission, Psyllids, *Trioza*

## Abstract

The apple proliferation (AP) disease is spread mostly by two psyllids. Each species plays a predominant role as AP vector that changes regionally. Thus, there is an urgent need to identify the AP vectors in each region where the disease is present. This research aimed at identifying the psyllid community in apple orchards from Asturias (NW Spain) and studying their possible role in AP transmission. Yellow sticky traps were used to monitor psyllid community in five cider-apple orchards during 2 years. 3678 individuals belonging to 22 species were identified. We confirmed the presence of the two known vectors, *Cacopsyllapicta* and *Cacopsylla melanoneura*, although they occurred in relatively low numbers (2.1 and 0.7 % of total catches, respectively). Most collected psyllids are not supposed to use apple as host, and their occurrence is likely favoured by landscape structure and an insect-friendly management. Phytoplasma detection was performed by squash-capture real-time PCR. The pathogen was detected in six species (*Cacopsylla crataegi, Cacopsylla mali, Ctenarytaina spatulata,Ctenarytaina eucalypti* and the two known AP vectors). Based on abundance and AP-detection rate *C. picta* is likely the main species spreading AP in our region. However, the low density of the known vectors does not match the widespread and high tree damage level observed in Asturias. The discovery of other four psyllid species carrying the phytoplasma reveals that our knowledge on the potential vectors is limited and that more research is clearly needed to unravel the role of the psyllid fauna in disease transmission in our orchards.

## Background

The apple proliferation (AP), induced by ‘*Candidatus* Phytoplasma mali’, is a serious disease that causes significant economic losses to apple growers. The multiplication and spread of phytoplasmas in apple trees is accompanied by the appearance of different symptoms (Kartte and Seemüller 1988; Zimmermann et al. 2015). The most characteristic symptom allowing a reliable identification of the infected plants is the witches’ brooms caused by the loss of apical dominance of shoots and the proliferation of axillary buds. Other symptoms include foliar reddening, enlarged stipules, or smaller fruits with poor taste, what reduces significantly their marketability. The disease is widespread in Europe and neighbouring regions, with a highest economic impact occurring in Germany and northern Italy (Foissac and Wilson 2010). Annual losses due to AP in these two countries have been estimated in € 125 million (Strauss 2009).

Apple proliferation symptoms have also been frequently observed in the last decade in apple orchards in Asturias, in the North coast of Spain. This region has long tradition in cider-making, and most of the 10,000 ha of apple orchards are cultivated almost exclusively to produce cider-apples. The majority of the surface devoted to apple production is occupied by traditional extensive orchards with big trees grown on seedling rootstocks. Since the early 1990s, an increasing percentage of these old orchards have been substituted by new semi-intensive ones with trees growing on semi-dwarfing rootstocks (Dapena et al. 2005). Those orchards are planted with selected local cultivars tolerant to several common apple diseases (scab, canker, powdery mildew). The use of pesticide in both situations is very low, even null. Sprays are mainly made with narrow-spectrum insecticides against the codling moth (*Cydia pomonella* L.) or the rosy apple aphid (*Dysaphis plantaginea* Passerini). Such situation of low pressure of pesticide use potentially allows high abundance and diversity of insects in orchards (Miñarro et al. 2005, 2009). The immersion of those orchards in an agricultural landscape characterized by a mosaic of small and different-land-use plots separated by hedgerows, as well as the occurrence of a permanent rich floral groundcover, also favour insect abundance and richness (Miñarro and Prida 2013; Rosa García and Miñarro 2014).

Phytoplasmas are obliged parasites which require a host plant as a reservoir of the disease and an insect host that contributes to spread the pathogen from an infected plant to a healthy one (Weintraub and Beanland 2006; Firrao et al. 2007; Alma et al. 2015). Since phytoplasmas are phloem-limited, only phloem-feeding insects can potentially acquire and transmit the pathogen (Weintraub and Beanland 2006). The available knowledge on the insect vectors of apple proliferation was summarized by Jarausch and Jarausch (2010). Different psyllid species belonging to the genus *Cacopsylla* (Hemiptera: Psyllidae) are considered the main vectors responsible for the transmission of European fruit tree phytoplasmas (Alma et al. 2015). Among them, *Cacopsyllapicta* (Foerster) and *Cacopsylla melanoneura* (Foerster) are the known vectors of AP. Whereas *C.picta* is the known vector in Germany and NE Italy (Jarausch and Jarausch 2010), *C. melanoneura* was only confirmed as AP vector in Italy (Tedeschi and Alma 2004; Mayer et al. 2009; Tedeschi et al. 2012). *Cacopsylla picta* is monophagous on *Malus* spp., whereas *C. melanoneura* is oligophagous on Rosaceae such as *Crataegus*, *Malus* and *Pyrus* (Ossiannilsson 1992). Both species are univoltine and overwinter as an adult on conifers. Both generations, the overwintered and the new adults, can transmit AP (Tedeschi and Alma 2004; Jarausch et al. 2011), that is, *C.picta* and *C. melanoneura* are able to transmit the phytoplasma during the entire period when they are on apple trees.

‘*Candidatus* Phytoplasma mali’ has also been detected in other *Cacopsylla* species although their potential to transmit the disease is debatable (Tedeschi et al. 2009; Baric et al. 2010). Rosa García et al. (2014) also found the phytoplasma in two exotic eucalypt psyllid pests although their role as vectors remains unknown. Finally, the leafhopper *Fieberiella florii* (Stal) can also transmit AP in experimental conditions (Tedeschi and Alma 2006) although its role in the spread of AP is questionable given the low density of the species in apple orchards, at least in NW Italy (Tedeschi and Alma 2006).

There is still no treatment to cure AP-infected trees and thus the known possibilities to control the disease include preventing the pathogen spread by planting healthy material, uprooting diseased plants and acting against vectors (Baric et al. 2010). Regarding vector control, there is consequently an urgent need to identify which are the AP vectors in each region where the disease is present (Alma et al. 2015).

In Asturias, the available knowledge is limited to a preliminary survey focused on a single orchard (Laviña et al. 2011) which detected very low numbers of the known vectors (one specimen of *C. picta* and 12 of *C. melanoneura*), but no information was given on AP infection. In the Basque Country, close to Asturias, *C. picta* has been proposed as the main AP vector, according to its abundance and detection rate (Batlle et al. 2012). The widespread presence of AP symptoms in Asturias induced us to conduct a more-in-depth study on a larger number of orchards from different areas in the region to identify the psyllid community in the apple orchards of Asturias and to search for AP in the psyllids, with the main aim of analyzing their possible role in phytoplasma transmission.

## Methods

### Site description

**Fig. 1:**
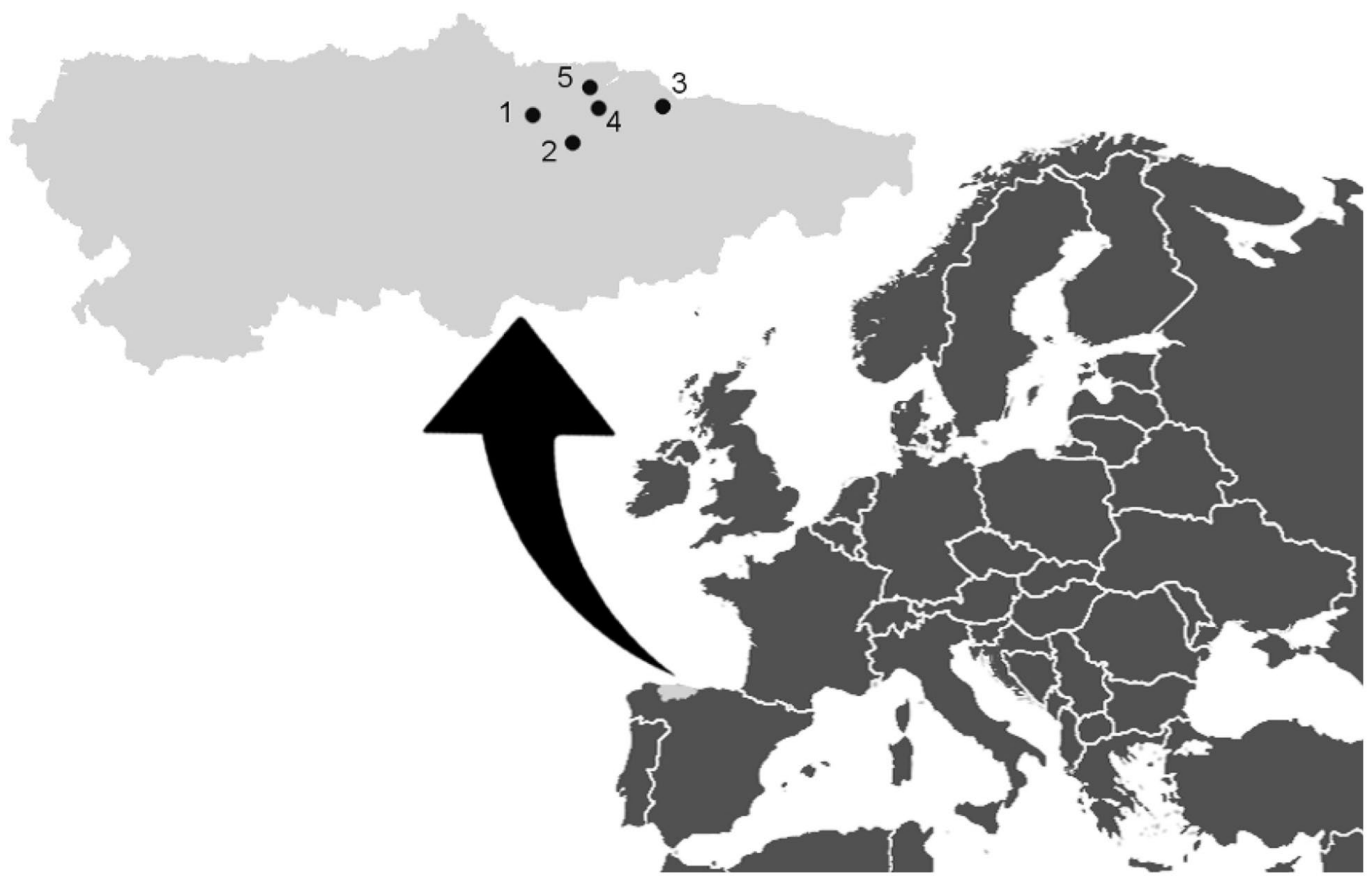
Map indicating the location of the five orchards where psyllids were sampled. (*1* Siero, *2* Nava, *3* Colunga, *4* Villa 1, *5* Villa 2)

Psyllid (superfamily Psylloidea) populations were monitored between February 2011 and December 2012 in five cider-apple orchards (Colunga, Siero, Nava, Villa 1 and Villa 2) located in Asturias, NW Spain (Fig. 1). A description of the experimental orchards is given in Table 1. At least seven different local cultivars were simultaneously grown in each orchard, as typically in cider orchards in this region. Asturias has a temperate oceanic climate with a fairly evenly spread rainfall over the year usually exceeding 1000 mm. Meteorological data comprising daily rainfall as well as temperatures in the sampling period are shown in Rosa García et al. (2014). The occurrence of the AP disease was visually confirmed in all the orchards by the presence of witches’ brooms, although the number of symptomatic trees was not recorded.

**Table 1:** Orchard description (IFP- Integrated Fruit Production)

Orchard	Orchard type	Guidelines	Year of plantation	Area (m^2^)	Altitude (m)	Coordinates
Siero	Semi-intensive	IFP	1996	7600	333	N 43° 26′ 03′′–W 5° 36′ 47′′
Nava	High-stem trees	Organic	1997	11,600	258	N 43° 21′ 57′′–W 5° 29′ 31′
Colunga	Semi-intensive	IFP	1996	8000	40	N 43° 28′ 52′′–W 5° 14′ 57′′
Villa 1	Semi-intensive	Organic	1994	4200	2	N 43° 28′ 44′′–W 5° 26′ 19′′
Villa 2	Semi-intensive	Organic	1989	8100	2	N 43° 28′ 34′′–W 5° 26′ 30′′

### Insect sampling and species identification

Three yellow sticky traps (200 mm × 250 mm, Econex ^®^) were randomly installed in each site to identify and quantify the abundance/activity of adult psyllid fauna. The traps were hanged in the trees and replaced weekly. Psyllids were identified after examining morphological features, emphasizing in male and female terminalia, according to Hodkinson and White (1979) and Ossiannilsson (1992). The specimens were mostly alive when removed from the sticky traps and they were immediately transferred to 100 % ethanol and stored at 5 °C until processed for AP detection.

### Detection of ‘*Candidatus* Phytoplasma mali’ in psyllids

Phytoplasma detection in psyllids was performed by squash-capture real-time PCR. Single psyllids were squashed on paper with the rounded end of an Eppendorf tube. Pieces of squashed samples were inserted into Eppendorf tubes and 100 ml of Triton X-100 0.5 % were added (Olmos et al. 1999), incubated at 95 °C for 10 min, vortexed and placed on ice. Five microliters of each extract were directly used for the PCR assays. TaqMan assays for real-time PCR were performed in ABI Prism 7300 Sequence Detection System software (Applied Biosystems) according to Aldaghi et al. (2007) with a few modifications. The reaction cocktail contained 2× TaqMan^®^ Gene Expression Master Mix (Applied Biosystems), 10 μM primer qAP-16S-F (5′ CGA ACG GGT GAG TAA CAC GTA A 3′), 10 μM primer qAP-16S-R (5′ CCA GTC TTA GCA GTC GTT TCC A 3′), 5 μM TaqMan probe AP-MGB (5′ FAM-CTG CCT CTT AGA CGA GG MGB 3′) and 5 μl of extracted DNA targets from the immobilized samples. Real-time PCR protocol consisted in an initial denaturation phase of 10 min at 95 °C followed by 45 cycles of amplification (95 °C for 15 s and 64 °C for 1 min). Data acquisition and analysis were performed with the ABI Prism 7300 software. Each sample was analyzed as two technical replicates to corroborate the positive results.

### Data analyses

Kruskal–Wallis tests were used to evaluate the among-site differences in psyllid abundance. χ^2^-tests were used to compare the number of males and females in each species. χ^2^-tests were also used to compare between-year differences in psyllid abundance. Statistical analyses were performed with SPSS (IBM SPSS statistics version 19.0.0).

## Results

### Psyllid community in apple orchards

A total of 3678 individuals belonging to 22 species were identified (Table 2). The number of species per orchard ranged from 15 to 19 (Table 2). *Ctenarytaina spatulata* Taylor and *Ctenarytaina eucalypti* (Maskell) were respectively the first (59.7 % of total catches) and the fifth (2.8 %) most abundant species. The results concerning these two species have been previously reported as a singular case because they are exotic pests whose common hosts were supposedly limited to *Eucalyptus* species and this was the first time in which they were recorded feeding on apple (Rosa García et al. 2014). Therefore, results regarding these species are not repeated in the present paper. In addition, only results for species with more than 25 specimens are shown here.

**Table 2:** Abundance of psyllid species in each apple orchard and year

Species	Total	%	Orchard					Year^a^		Effects^b^	
			Colunga	Nava	Siero	Villa 1	Villa 2	2011	2012	Orchard	Year
*Ctenarytania spatulata*	2195	59.7	595	217	555	429	399	1289	906	***	***
*Cacopsylla mali*	717	19.5	–	29	687	1	–	445	272	***	***
*Cacopsylla visci*	164	4.5	10	9	118	18	9	91	73	***	ns
*Cacopsylla saliceti*	153	4.2	10	20	100	11	12	133	20	ns	***
*Ctenarytania eucalypti*	104	2.8	7	54	28	8	7	79	25	*	***
*Trioza urticae*	94	2.6	23	3	25	19	24	44	50	*	ns
*Cacopsylla picta*	79	2.1	7	31	24	3	14	30	49	**	*
*Trioza remota*	49	1.3	2	11	18	16	2	12	37	***	***
*Cacopsylla melanoneura*	27	0.7	10	4	8	2	3	13	14	ns	ns
*Trioza alacris*	18	0.5	7	–	7	3	1	3	15	*	**
*Cacopsylla spp.*	17	0.5	6	1	8	1	1	10	7	ns	ns
*Aphalara polygoni*	12	0.3	1	2	4	3	2	9	3	ns	ns
*Homotoma ficus*	10	0.3	5	–	4	1	–	8	2	*	ns
*Cacopsylla crataegi*	8	0.2	–	7	1	–	–	2	6	**	ns
*Cacopsylla pyricola*	8	0.2	1	2	4	1	–	5	3	ns	ns
*Cacopsylla pulchra*	7	0.2	2	1	3	–	1	4	3	ns	ns
*Cacopsylla pruni*	5	0.1	1	2	1	–	1	–	5	ns	*
*Acizzia acaciaebaileyanae*	4	0.1	–	1	3	–	–	3	1	ns	ns
*Trioza flavipennis*	3	0.1	–	–	–	2	1	3	–	ns	ns
*Cacopsylla sorbi*	2	0.1	1	–	–	–	1	–	2	ns	ns
*Cacopsylla affinis*	1	0	–	–	–	1	–	1	–	ns	ns
*Psyllopsis fraxini*	1	0	–	–	1	–	–	–	1	ns	ns
Total	3678		688	394	1599	519	478	2184	1494	***	***
%			18.7	10.7	43.5	14.1	13	59.4	40.6		
# of species	22		16	16	19	16	15	19	20		

Differences in abundance due to orchards and years are indicated by asterisks

^a^ 45 weeks in 2011 and 51 weeks in 2012

^b^ ns—no significant

* p<0.05, ** p<0.01, *** p<0.001

Among the rest of psyllids, a total of 12 *Cacopsylla* species were collected. Apple-dwelling *Cacopsylla mali* (Schmidberger) was the most abundant one (19.5 % of total catches), although it was mainly recorded in one orchard (95.8 % of catches) and it was absent in two. *Cacopsylla visci* (Curtis) (4.5 %) and *Cacopsylla saliceti* (Foerster) (4.2 %) followed *C. mali* in abundance. The two known AP vectors, *C. picta* and *C. melanoneura* were detected in all the orchards, although their relative occurrence was low (2.1 and 0.7 % of total catches, respectively). Two *Trioza* species, *T. urticae* (L.) (2.6 %) and *T. remota* Foerster (1.3 %) were also among the psyllid species with more than 25 specimens.

There were significant differences among orchards in total psyllid abundance as well as in the abundance of all of the most abundant psyllids but one, *C. saliceti* (Table 2). The total psyllid catches were higher in 2011 than in 2012 despite the sampling periods lasted 45 weeks in 2011 and 51 weeks in 2012 (Table 2). The catches of four of the five most abundant species were also higher in 2011 than in the next year (Table 2).

Only the catches of the two most abundant species were sex-biased: *C. spatulata* catches were female-biased (60.7 % of females; χ^2^ = 100.21; p < 0.001) and *C. mali* catches were male-biased (73.5 % of males; χ^2^ = 158.36; p < 0.001).

**Fig. 2:**
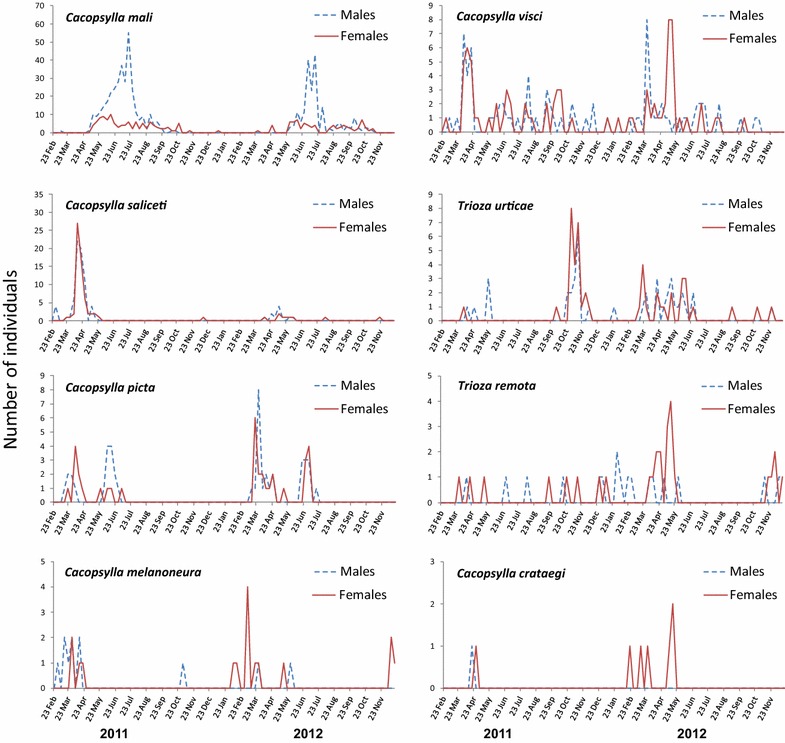
Population dynamics of psyllids (data from five orchards pooled) from 23 February 2011 to 19 December 2012. Data are shown for male and female individuals of the most abundant species (see Table 2) and/or those showing AP-positive specimens (see Table 3)

Population dynamics of the most abundant psyllids as well as those carrying phytoplasma are shown in Fig. 2. Most of those species showed a rather similar phenological pattern in both years. *Cacopsylla mali* was detected from spring to autumn, reaching a density peak in mid-summer. Curiously, only males contributed to that peak. *Cacopsylla visci* was detected almost continuously, although a small density peak was observed in spring. *Cacopsylla saliceti* only showed a clear density peak in the spring of 2011, whereas such peak was more subtle in 2012 due to the lower catches. *Cacopsylla picta* showed two peaks every year, one in March–April and other around June. *Cacopsylla melanoneura* appeared at the end of winter, peaking early in the spring. The catches of *Cacopsylla crataegi* (Schrank) were low and occurred in spring, from February to May. Finally, neither a clear nor a repetitive pattern was appreciated in the case of the most abundant *Trioza* species.

### Detection of ‘*Candidatus* Phytoplasma mali’ in psyllids

Phytoplasma detection was performed for the eight most abundant species and the presence of the pathogen was confirmed in six of them (Table 3). The highest detection rate (10.0–16.7 %) was found in four *Cacopsylla* species (*C. crataegi*, *C. picta*, *C. melanoneura* and *C. mali*). On the contrary, the detection rate in the two exotic *Ctenarytaina* species was below 3 %. AP-positive males were detected in all the six species whereas positive females were detected only in two of them (Table 3). The global detection rate was higher for males (7.1 %) than for females (2.1 %). The spatial distribution of the AP-positive specimens is unclear because the abundance and consequently the number of evaluated specimens varied widely among sites (Tables 2 and 3). Nevertheless, AP-positive individuals were detected in all the orchards, ranging from 2.8 to 6.7 % (Table 3). In addition, in one orchard four AP positive species were confirmed whereas in three orchards only one AP-positive species was detected (Table 3). AP-positive individuals were found from March to October, depending on the species, being globally higher in March and from June onwards (Table 4).

**Table 3:** Results of phytoplasma detection [no positive detections/total number of analyzed individuals (%)] for the most abundant species according to sex and orchard

Species	Total	Ct* (mean ± SE) [max−min]	Sex		Orchard				
			Male	Female	Colunga	Nava	Siero	Villa 1	Villa 2
*Cacopsylla crataegi*	1/6 (16.7)	34.95	*1/1 (100.0)*	0/5	–	*1/5 (20.0)*	0/1	–	–
*Cacopsylla picta*	9/68 (13.2)	28.44 ± 1.47	*7/37 (18.9)*	*2/30 (6.7)*	0/6	*4/30 (13.3)*	*3/19 (15.8)*	0/1	*2/12 (16.7)*
*Cacopsylla melanoneura*	1/10 (10.0)	[23–35] 33.57	*1/2 (50.0)*	0/8	0/1	*1/3 (33.3)*	0/3	0/2	0/1
*Cacopsylla mali*	6/60 (10.0)	34.35 ± 0.22 [34–35]	*4/37 (10.8)*	*2/23 (8.7)*	–	0/6	*6/54 (11.1)*	–	–
*Ctenarytaina spatulata*	2/75 (2.7)	32.00 ± 1.00 [31–33]	*2/38 (5.3)*	0/37	0/13	*1/11 (9.1)*	0/20	*1/18 (5.6)*	0/13
*Ctenarytaina eucalypti*	1/73 (1.4)	35.02	*1/36 (2.8)*	0/37	*1/6 (16.7)*	0/36	0/19	0/7	0/5
*Cacopsylla saliceti*	0/60	–	0/40	0/20	0/6	0/10	0/33	0/4	0/7
*Cacopsylla visci*	0/60	–	0/33	0/27	0/4	0/3	0/48	0/2	0/3
Total	20/412 (4.9)		16/224 (7.1)	4/187 (2.1)	1/36 (2.8)	7/104 (6.7)	9/197 (4.6)	1/34 (2.9)	2/41 (4.9)

Positive results in italics

Ct* mean threshold cycle by species

**Table 4:** Results of phytoplasma detection (nº positive detections /total of analyzed individuals) according to the month

Species	Jan	Feb	Mar	Apr	May	Jun	Jul	Aug	Sep	Oct	Nov	Dec
*Cacopsylla crataegi*	–	–	0/1	*1/2*	0/3	–	–	–	–	–	–	–
*Cacopsylla picta*	–	–	*6/23*	*1/16*	0/3	*1/22*	*1/4*	–	–	–	–	–
*Cacopsylla melanoneura*	–	0/2	*1/6*	0/1	0/1	–	–	**–**	**–**	**–**	–	–
*Cacopsylla mali*	–	–	–	–	0/5	*3/15*	0/14	*1/10*	*1/10*	*1/5*	0/1	–
*Ctenarytaina spatulata*	0/5	0/6	0/5	0/7	0/8	*1/6*	0/1	*1/9*	0/6	0/10	0/8	0/4
*Ctenarytaina eucalypti*	0/1	–	0/7	0/28	*1/18*	0/1	–	–	–	–	0/10	0/8
*Cacopsylla saliceti*	–	–	0/1	0/43	0/15	–	–	0/1	–	–	–	–
*Cacopsylla visci*	0/3	0/2	0/2	0/8	0/13	0/10	0/3	0/6	0/5	0/5	0/1	0/2
Total	0/9	0/10	*7/45*	*2/105*	*1/66*	*5/54*	*1/22*	*2/26*	*1/21*	*1/20*	0/20	0/14
%	0	0	15.6	1.9	1.5	9.3	4.5	7.7	4.8	5.0	0	0

Positive results in italics

## Discussion

The present research provides the first comprehensive approach to the psyllid fauna in apple orchards in Asturias, NW Spain, and allowed us to identify the species carrying the phytoplasma, which are consequently candidates to be AP vectors in this region. Among them, we confirmed the presence of *C.picta* and *C. melanoneura*, the two known AP vectors in other European regions (Jarausch and Jarausch 2010).

*Cacopsylla picta* and *C. melanoneura* use apple as primary hosts, although *C. melanoneura* can also reproduce on hawthorn and pear, and both species use conifers for overwintering (Ossiannilsson 1992). The phenology of both psyllids is synchronized with apple phenology, and it is probably mediated by temperature (Tedeschi et al. 2012): at the end of winter re-migrant adults move from the overwintering sites to apple trees to reproduce and lay eggs before or coinciding with bud break (Tedeschi et al. 2002, 2012; Jarausch et al. 2011). The new generation leaves the host plant in summer and migrates to the overwintering hosts. In the current study, the adult *C. melanoneura* arrived to and left the apple tree earlier than *C. picta* did. This trend matches the previous knowledge for the phenology of these species (Tedeschi et al. 2002, 2012; Jarausch et al. 2011).

The actual risk of transmission of a pathogen to a crop under field conditions could be estimated by the vector intensity (Irwin and Ruesink 1986), which is determined as the final product of two factors: the vector activity (number of insects visiting the crop) and the vector propensity (probability of a vector transmitting a pathogen under field conditions). The number of insects carrying the pathogen can also influence the second factor. Accordingly, each species seems to play a predominant role in different regions: whereas *C. picta* is the main AP vector in Germany and NE Italy, *C. melanoneura* is the main vector in NW Italy (Jarausch and Jarausch 2010 and references therein). Nevertheless, Tedeschi et al. (2012) recently demonstrated the importance of *C. melanoneura* in spreading AP also in NE Italy. In our case, both species were collected in all the study sites. *Cacopsylla picta* catches were three times those of *C. melanoneura*, being clearly higher in three of the orchards, contrarily to the previous observation of Laviña et al. (2011) in one site located also in Asturias, who reported one specimen of *C. picta* and 12 of *C. melanoneura* in the sampled orchard. In any case, the abundance of these species in our orchards was very low compared to the other psyllid species but it also differs from other studies (e.g. Tedeschi et al. 2003; Jarausch et al. 2009). In our two-year sampling we recorded only a total of 79 and 27 specimens of *C. picta* and *C. melanoneura*, respectively. For instance, Tedeschi et al. (2012) recorded up to eight specimens of *C. melanoneura* per branch at a single sampling event using a beating method.

Both psyllids are highly efficient vectors of AP and they can transmit the phytoplasma during the entire period when they are on apple trees, although transmission trials confirmed that the overwintered generation would be more effective than the new adults (Tedeschi and Alma 2004; Jarausch et al. 2011). Phytoplasma acquired by the new generation could have a very low titre and maybe the latency period is not yet completed before migration to conifers (Tedeschi et al. 2003). This would explain such generation differences as well as the highest detection rate in the overwintering generation. For *C. melanoneura*, Tedeschi et al. (2003) found 2.8–3.6 % AP-positive specimens in the overwintering generation versus 0–0.8 % in the spring one. The low captures of *C. melanoneura* prevented a similar comparison in our study. Otherwise, we observed a similar pattern in *C. picta*: 7 of 39 (17.9 %) of the specimens collected in March and April were infected *versus* only 2 of 26 (7.7 %) specimens from June to July. On the other hand, Jarausch et al. (2011) reported a similar phytoplasma titre along time in overwintering *C. picta*, confirming that the phytoplasma overwinters in the insect.

Our AP detection rate for *C. picta* (13.2 %) is similar to that previously obtained in other European regions [≈10 % in a German-Swiss-Italian study (Jarausch et al. 2011) or 11.1 % in NE Italy (Baric et al. 2010)]. On the contrary, our AP-detection rate for *C. melanoneura* (10.0 %) is markedly higher than the one reported in other regions where this species is not an AP vector [0.09 % in Germany (Mayer et al. 2009) or 0.6 % in NE Italy (Baric et al. 2010)] but also higher than where *C. melanoneura* transmits the phytoplasma [3.6–6.5 % for the overwintering generation and less than 0.8 % in the spring one in NW and NE Italy (Tedeschi et al. 2003, 2012)].

High infection rates have been correlated with transmission ability both at specific and at generation level (Tedeschi and Alma 2004; Mayer et al. 2009; Jarausch et al. 2011). Accordingly, both species, *C. picta* and *C. melanoneura*, might be able to transmit AP in NW Spain, but transmission trials are needed to confirm it (e.g. Tedeschi and Alma 2004; Jarausch et al. 2011).

*‘Candidatus* Phytoplasma mali’ is a phloem-restricted pathogen and thus insects feeding on phloem could acquire the phytoplasma. AP-positive individuals have been reported for other psyllids: *C. mali* (Baric et al. 2010) and *Cacopsylla peregrina* (Foerster) (Tedeschi et al. 2009), as well as for aphids (Cainelli et al. 2007). However, transmission has not been proved for these species. We have detected AP-positive specimens in *C. mali* and also in *C. crataegi*. To our knowledge this is the first time that AP was detected for *C. crataegi*. Finally, as previously reported (Rosa García et al. 2014) we found positive specimens in the two exotic psyllids, *C. spatulata* and *C. eucalypti*. These two eucalypt pests were present in all the orchards and were abundant, especially the former, although their infection rate was low (<3 %) in both cases. *Cacopsylla mali* was also abundant and showed important infection rates (10 %), but it was not detected in all orchards. *Cacopsylla crataegi* showed the highest detection rate (16.7 %) albeit its density was very low (eight specimens in just two orchards).

In any case, the occurrence of AP-positive specimens indicates that all these species reach the phloem of infected trees, although the acquisition of a phytoplasma does not necessarily mean that the insect is a vector. As a previous step to transmission, the phytoplasma should multiply itself inside the insect and reach the salivary glands to be transmitted in a new feeding event on a healthy plant (Weintraub and Beanland 2006). Therefore, experimental transmission trials are required again to prove that a phytoplasma-positive insect is a vector. As a first step before transmission trials, quantitative real-time PCR is a useful tool to have a first indication between non-vectors, which have just acquired the phytoplasma by sucking, and real vectors which have multiplied the phytoplasma (Jarausch and Jarausch 2010). Furthermore, in the field, phloem-restricted pathogens are transmitted efficiently only by colonizing species, and from an epidemiological point of view, the transmission of a persistent phloem-restricted plant pathogen by non-colonizing species (most species in our study) is very unlikely to occur (Irwin et al. 2007).

A thorough and extensive sampling (7 years, 50 orchards) in Germany, France and Switzerland reported 25 psyllid species inhabiting apple orchards (Jarausch et al. 2009). With a considerably lower effort (2 years, 5 orchards) we collected 22 species, varying the number of species per site between 15 and 19. Such a high richness is likely a consequence of the low pesticide input in our cider-apple orchards as well as the high plant richness in the orchards and their surroundings (Miñarro and Prida 2013; Rosa García and Miñarro 2014), since most of the collected psyllids have not apple among their hosts (Ossiannilsson 1992; Jarausch et al. 2009). In fact, only three species, *C. mali*, *C. picta* and *C. melanoneura*, have apple as a host (Ossiannilsson 1992). They are the most abundant psyllids in apple orchards (Jarausch et al. 2009), although not in our study. The occurrence and density of the *Ctenarytaina* species are easily explained by the wide distribution of eucalypt crops in the surroundings of the orchards (Rosa García et al. 2014). The third most abundant species, *C. visci*, has mistletoe (*Viscum album* L.) as host (Hansen and Hodkinson 2006), and this parasitic plant frequently grows on apple in our region. The other abundant species, *C. saliceti*, *T. urticae* and *T. remota* Foerster, live on *Salix, Urtica* and *Quercus*, respectively (Ossiannilsson 1992), all of which can be either present in the orchards or in nearby habitats.

Since there is no therapy available to cure infected trees, disease control should rely on prevention. Therefore, monitoring the vectors should be a crucial step for control, and this could be implemented with forecasting models (Tedeschi et al. 2012). However, the high species richness detected in our orchards, as well as the relative low densities of the known vectors, impose serious constraints to reliably monitor the presence of vectors in the orchards as morphological identification is problematic and time-consuming. Fortunately, recently developed molecular tools allow a reliable identification of *Cacopsylla* species inhabiting orchards (Oettl and Schlink 2015), facilitating monitoring. On the other hand, chemical control of vectors in our region is difficult. First, because of the difficulty to monitor such low vector densities. And second because the management of most potential pests in our orchards is based on biological control (e.g. Miñarro et al. 2005) and the use of broad-spectrum insecticides against vectors would probably disrupt such control increasing the appearance of secondary pests. Alternative methods, such as mass trapping with phytopathogen-induced plant alomones (Eben and Gross 2013), would be welcome.

## Conclusion

We report new data related to new epidemiological situations as a result of the particular environmental situations in apple production in NW Spain. Our cider-apple orchards are inhabited by an abundant and rich psyllid community, including the two so far known AP-vectors as well as exotic species and others which are not supposed to use apple as host. Total captures of *C. picta* (79) were clearly higher than those of *C. melanoneura* (27) and, although both detection rates were rather similar (around 10 %), rate of *C. picta* is more significant because the percentage of positive specimens was more regular among orchards. Thus, *C. picta* is likely the putative vector in our region, although a contribution of *C. melanoneura* or other species cannot be discarded. Anyway, the wide distribution of the disease and the high rate of symptomatic trees in our region contrast with the low density of the known vectors. Multiplication of infected plant material and root bridges between nearby trees could be also contributing to spread the phytoplasma among and inside orchards, respectively (Ciccotti et al. 2007). In any case, the discovery of other four psyllid species, apart from the known vectors, carrying the phytoplasma reveals that our knowledge on the potential vectors is quite limited and goes far beyond the typical ones. More research is clearly needed to unravel the role of the psyllid fauna inhabiting our orchards in the transmission of the phytoplasma as well as to evaluate the influence of the surrounding landscape.
